# Tauroursodeoxycholic acid combined with selenium accelerates bone regeneration in ovariectomized rats

**DOI:** 10.1007/s10856-024-06803-0

**Published:** 2024-10-15

**Authors:** ZhouShan Tao, Min Yang, Cai-Liang Shen

**Affiliations:** 1 Department of Orthopedics, The First Affiliated Hospital of Wannan Medical College, Yijishan Hospital, No. 2, Zhe Shan Xi Road, Wuhu, 241001 Anhui PR China; 2 Anhui Province Key Laboratory of Non-coding RNA Basic and Clinical Transformation, No. 2, Zhe Shan Xi Road, Wuhu, 241001 Anhui PR China; 3 Department of Spinal Surgery, The First Affiliated Hospital of Anhui Medical University, Hefei, PR China

## Abstract

More recently, increased studies have revealed that antioxidants can cure osteoporosis by inhibiting oxidative stress. Tauroursodeoxycholic acid (TUDCA) and Selenium (Se) have been confirmed to possess potent anti-oxidative effects and accelerate bone regeneration. In addition, very little is currently known about the effects of a combination with Se and TUDCA on bone defects in osteoporotic states. We, therefore, aimed to assess the protective effect of combination with Se and TUDCA on bone regeneration and investigate the effect and underlying mechanisms. When MC3T3-E1 was cultured in the presence of H_2_H_2_, Se, TUDCA and Se/TUDCA therapy could increase the matrix mineralization and promote expression of anti-oxidative stress markers in MC3T3-E1, while reducing intracellular reactive oxygen species (ROS) and mitochondrial ROS levels. Meanwhile, silent information regulator type 1 (SIRT1) was upregulated in response to Se, TUDCA and Se/TUDCA exposures in H_2_H_2_ treated-MC3T3-E1. In the OVX rat model, Se, TUDCA and Se/TUDCA showed a clear positive effect against impaired bone repair in osteoporosis. The results above demonstrate that Se/TUDCA exhibits superior efficacy in both cellular and animal experiments, as compared to Se and TUDCA. In conclusion, combination with Se and TUDCA stimulates bone regeneration and is a promising candidate for promoting bone repair in osteoporosis.

**Graphical abstract:**

**Figure:**
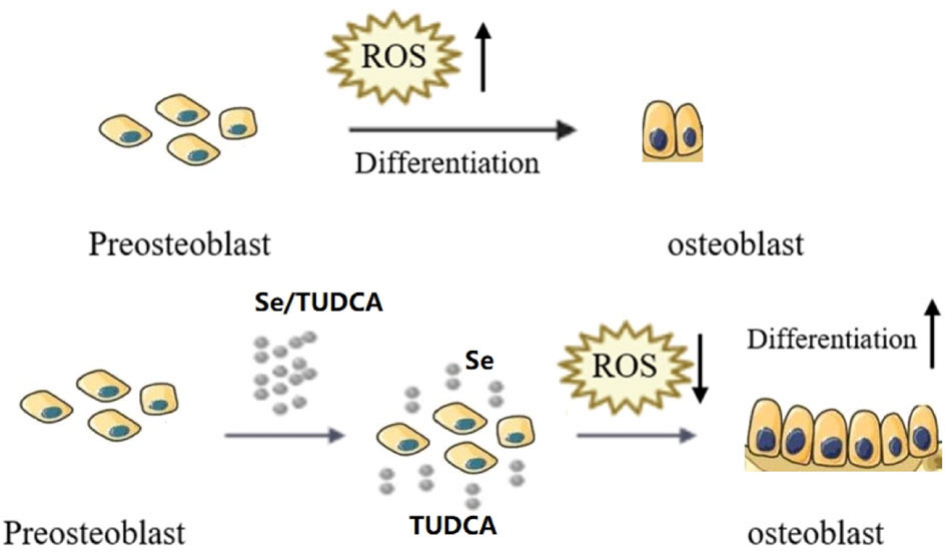
The release of TUDCA and Se during the degradation of Se/TUDCA can improve the local bone repair ability. At the same time, it can also inhibit cell ROS, and ultimately greatly promote local bone repair.

## Introduction

Osteoporotic bone defects can occur in any part of the skeletal system, including the spine, legs, arms, ribs or skull [1–3]. The condition can have serious implications for a patient’s overall health and well-being [4]. Options could include conservative measures such as pain management and physical therapy, surgical procedures to repair or replace affected bone, or even bone grafting techniques to promote new bone growth [5]. Although minimally invasive techniques for fractures and bone defects provide great benefits to patients, reconstruction of osteoporotic bone defects remains challenging. Recently, the incidence of osteoporotic fractures has increased with the aging population [6]. However, osteoporotic fractures are complicated with delayed union or non-union reported to be as high rates (10–20%) [7]. This non-union especially in most important sites of fracture including hip and spine causes disability and subsequent loss of mobility to patients and, therefore, raised a number of health issues [8]. Therefore, osteoporotic bone defects are a complex and challenging medical condition that require specialized care and treatment. The management of osteoporotic bone defects has emerged as a challenging issue that necessitates collaborative efforts between clinical orthopedic practitioners and patients for resolution.

In order to prevent bone defect delayed union and promote bone repair, a number of strategies have been successfully implemented, including autologous grafts and supplementation with *bone morphogenetic protein*-*2* (*BMP*-*2*) [9, 10]. Although autologous bone transplantation is considered the gold standard in the treatment of bone defects, however, it poses potential surgical complications for harvesting autogenous bone grafts and donor sites [11]. Moreover, osteoporotic bone defects are regularly seen in older patients with many chronic diseases, and the risk of the procedure is increased, posing a great challenge to both surgical patients and clinicians. In fact, BMP-2 has been reported in preclinical and clinical studies to enhance bone repair. More recently, however, many complications have been reported in various studies such as new onset of cancer heterotopic ossification and retrograde ejaculation [12, 13]. Tauroursodeoxycholic acid (TUDCA) is a hydrophilic nontoxic bile acid produced from intestinal bacteria, which is regarded as an *endoplasmic reticulum* (*ER*) *stress inhibitor* [14]. Several studies have proven that TUDCA has been applied to intervene in human mesenchymal stem cells to suppress adipogenesis and angiogenesis promotion [15, 16]. It has previously been demonstrated that TUDCA enhances osteoblast differentiation and promotes the formation of calcified nodules in vitro [17]. In a previous spinal fusion study, TUDCA showed the same underlying osteogenesis as BMP-2, inducing bone remodeling and bone formation [18]. Selenium (Se), an essential trace element, plays a crucial role in human health and well-being [19]. It is involved in a variety of physiological processes, including bone metabolism [20]. Additionally, Se is a key component of antioxidant enzymes that protects the body from oxidative stress, which can lead to bone loss and osteoporosis [21]. A deficiency in Se can, therefore, have negative implications for bone health, increasing the risk of fractures and other bone-related disorders [22]. The local addition of trace amounts of Se in our previous study on bone defects has been found to enhance osteoporotic bone regeneration, thereby stimulating bone remodeling and formation [20, 21].

Taking inspiration from our previous study [21, 23], the combination of TUDCA and Se for the treatment of osteoporotic bone defects may yield more favorable outcomes compared to their individual use. However, to the best of our knowledge, there is a lack of such local studies using TUDCA and Se in combination for osteoporotic defect repair. Therefore, in the present study, we investigated the repair of TUDCA and Se in combination on a bone defect model in ovariectomized (OVX) rats.

## Material and methods

### Materials

In this study, collagen sponge (Wuxi Bedi Bioengineering Co., LTD) was used as an absorbable matrix scaffold for drug delivery. In addition, Na_2_SeO_3_ (Sigma-Aldrich, sodium selenite) and TUDCA (Wuhan Costan Biotechnology Co., LTD) were used as intervention factors to promote bone formation. In addition, the collagen sponge (25 mm × 10 mm × 1 mm) impregnated with Na_2_SeO_3_ (3 μg/30 μL) or/and TUDCA (300 μg/30 μL) solution acts as a filling scaffold for bone defect areas in OVX rats. The doses and route of local application of Se and TUDCA used in this study according to previous reports [18, 20].

### Surgical procedure and treatment

Healthy female Sprague Dawley (12-week-old, SD) rats are raised in a standard laboratory environment with standard diet and water and controlled room temperature (24 ± 2 °C). After adaptation for 1 week, 230∼280 g SD rats were used for establishing the standard hormone deficiency model through bilateral ovariectomy according to our previous reports [24, 25]. The bilateral ovaries of Sham rats were not removed. At 12 weeks after surgery, a rat femur defect model was built to explore the osteogenesis capacity of Se and TUDCA. Once the successful establishment of OVX osteoporosis model, femur defect drilling was performed on the femur condyles according to previous reports [24, 25]. Briefly, after routine disinfection and anesthetic, a 1 cm skin incision was made to expose the lateral condyle of the femur. A bicortical channel with a diameter of 2.0 mm was then drilled using a slow electric drill. Continuous irrigation with a saline solution was used to reduce thermal necrosis and flush out bone dust and debris during the establishment of bone defect model. For defects in the Se, TUDCA and Se/ TUDCA group, the defect site was filled with a collagen sponge containing Se, TUDCA or Se/TUDCA. After closing the incision, the rats were given antibiotics and analgesia for 3 days. After surgery for a bone defect, the rats were randomly divided into four groups: Control drilled defect group (Con, *n* = 10), Se treatment group (Se, *n* = 10), TUDCA treatment group (TUDCA, *n* = 10), and Se/TUDCA treatment group (Se/TUDCA, *n* = 10). Normal feeding patterns were maintained for 3 months after femur surgery, the treated rats from three groups were sacrificed, and the femurs were collected for follow-up testing and evaluation.

### Micro-computed tomography assessment

The new bone formation and bone trabeculae of the femur were scanned and evaluated by Micro-computed tomography assessment (Micro-CT, Skyscan 1176, Bruker Belgium SA) in different groups of rats as described in previous reports [21, 26, 27]. The scanning parameters are set as follows: 70 keV voltage; 114 µA, spatial resolution with 100–200 μm; slice thickness 120 μm, feed 60 μm and pixel size 60 μm. Bone trabecular parameters and bone mineral density (BMD) of distal femur were calculated using auto-calculated by computer. In parallel, new bone formation in the defect area was also scanned and evaluated by Micro-CT at 12 weeks after femur implantation. After reconstruction, these parameters were measured and compared among groups such as bone volume/tissue volume (BV/TV), trabecular thickness (Tb.Th), trabecular number (Tb.N), trabecular separation (Tb.Sp) and connective density (Conn.D) and BMD.

### Histological evaluation

The fixed femur specimens were decalcified in 10% ethylene diaminetetraacetic acid for 4 weeks and stained with HE to visualize the newly formed bone tissue in the defected femur and the changes of trabecular structure of distal femur according to previous reports [28–30]. Images were obtained using a biomicroscope (Ni-E, Nikon, Japan).

### Immunofluorescent analysis

The expression of Osteocalcin (OC) and Tartrate-resistant acid phosphatase (TRAP) in bone tissue of defect area was further investigated by examining bone sections prepared for histological analysis according to previous reports [29, 31]. Briefly, sections were deparaffinized, rehydrated and repaired by 0.05% trypsin at 37 °C for 30 min. Incubation with anti-OC (1:200, Boster Bio) and TRAP (1:200, Boster Bio) was performed at 4 °C for 10 h. DAB Kit (ZSGB-BIO Corporation, Beijing, China) was used to develop positive expression. Double immunofluorescence staining for SIRT1 and SOD2 was performed to detect levels of oxidative stress in bone tissue. After blocking with 5% (w/v) BSA for 1 h, the paraffin bone sections were incubated with anti-SIRT1 (1:100; Abcam, UK) and anti-Osteopontin (OPN, 1:100; Abcam, UK) overnight at 4 °C, and then incubated with fluorophore-conjugated secondary antibodies (1:200, Abcam, UK) for 1 h at 25 °C. Cell nuclei were stained with DAPI. For observation and image acquisition a fluorescence microscope was used, and the mean intensity of optical density was calculated semiquantitatively using Image Pro Plus software.

### Western blot

The levels of TRAP, OC, OPN and SIRT1 proteins in bone tissue at the site of bone defect were assessed using western blot analysis. Decalcified bone tissue was lysed with radio-immunoprecipitation assay buffer from Beyotime, and protein was collected after centrifugation. The concentration of each sample was determined using a bicinchoninic acid assay kit also from Beyotime. Subsequently, 20 μg of protein was separated by electrophoresis on a 10% SDS–PAGE gel and transferred onto polyvinylidene fluoride membranes obtained from Bio-Rad in Hercules, CA. To prevent nonspecific binding, the membranes were blocked with fat-free milk (5%) and then incubated overnight with antibodies against TRAP (dilution: 1:1000; Abcam), OC (dilution: 1:1000; Abcam), OPN (dilution: 1:1000; Abcam), SIRT1 (dilution: 1:1000; Abcam), as well as GAPDH (dilution: 1:2000). Afterward, HRP-labeled IgG (ab205718) at a dilution of 1:8000 from Abcam was applied for a 2-h incubation period. Following exposure to the BeyoECL Plus kit provided by Beyotime, the blots were analyzed using Quantity One software developed by Bio-Rad alongside GAPDH serving as an internal control for loading normalization.

### MC3T3-E1 cell experiments

In this study, murine osteoblast cell line MC3T3-E1 was used for the in vitro, which was purchased from Shanghai Cell Bank. MC3T3-E1 cells were seeded at normal medium (DMEM) with a density of 1 × 10^4^/ml and cultured with Se and/or TUDCA. Therefore, in vitro cell experiments were divided into three groups according to different intervention factors: normal medium (Control); normal medium plus sodium selenite (Se); normal medium plus TUDCA (TUDCA); normal medium plus Se and TUDCA; dosages used for Se (5 μg/l) or TUDCA (3 µg/l) in MC3T3-E1 cell experiments were chosen according to previous study [18, 21].

### Cell proliferation assay, alkaline phosphatase staining and alizarin red staining

The Cell Counting Kit 8 (CCK8, Dojindo, Japan) was used to assess MC3T3-E1 cell proliferation as described in previous reports [32, 33]. Briefly, 1 × 10^4^ MC3T3-E1 was seeded into each well of a 24-well plate and treated with Se or/and TUDCA for 72 h. Next, 10% CCK-8 solution was added to the culture medium for 4 h in 37 °C and examined the absorbance values by using a spectrophotometer (BioTek, Winooski, VT).

Afterward, the osteogenic direction of the MC3T3-E1 was induced by osteogenic differentiation medium (0.1 µM dexamethasone, 50 µM ascorbate-2 phosphate, 10 mM glycerophosphate (Sigma)). MC3T3-E1 was seeded onto 6-well plates with 1 × 10^5^ cells/well. When the cells reached ~70–80% confluence, osteogenic differentiation media were added and culture was carried out for 14 or 21 days. Following this, the treated cells were analyzed with alkaline phosphatase (ALP) staining and alizarin red (AR) staining to determine osteoblast differentiation. In brief, ALP staining was conducted using the NBT/BCIP Staining Kit (Beyotime Biotech, Shanghai, China) in accordance with the recommended protocol. Alizarin red (Beyotime, Shanghai, China) staining was utilized for visualizing calcium deposition as per the prescribed protocol. The areas of positive ALP staining and alizarin red staining were quantified utilizing NIH-Image J.

### Cellular immunofluorescence

MC3T3-E1 was seeded onto 6-well plates with 1 × 10^5^ cells/well. When the cells reached ~70–80% confluence, osteogenic differentiation media and 30 μM H_2_O_2_ were added and culture was carried out for 2 days. Then, the changes of oxidative stress were stained with 2′,7′-dichlorofluorescein diacetate (DCFH-DA, Sigma, St. Louis, MO) and MitoSOX™ Red mitochondrial superoxide indicator (MitoSOX™, Sigma, St. Louis, MO) according to the manufacturer’s instructions. SIRT1 staining (1:100; Abcam, UK) was performed to evaluate the density of *osteoblasts* in the callus area following a standard protocol. Measurement processes were performed as previously [21, 29].

### Quantitative real-time polymerase chain reaction (qRT-PCR) analysis

Total cellular RNA was extracted from the frozen bone around the implants using the Bone Tissue RNA Kit (Cowin Bioscience, Beijing, China) as described in previous reports [32, 34]. An amount of 800 ng of total RNA was reverse transcribed into cDNA using reverse transcriptase (M-MLV; Promega, Madison, WI, USA). qPCR was performed using with a SYBR Green qPCR Master Mix (EZBioscience, USA). Relative expression was calculated for each gene by 2^−△△^ CT method with GAPDH for normalization. Primer sequences used are shown in Table 1.

**Table 1:** Primers used in this study for quantitative real-time polymerase chain reaction (qRT-PCR)

Gene	Forward primer sequence (5′–3′)	Reverse primer sequence (5′–3′)
SIRT1	GCGGGAATCCAAAGGATAAT	CTGTTGCAAAGGAACCATGA
RANKL	CATCGGGTTCCCATAAAGT	CTGAAGCAAATGTTGGCGTA
SOD2	GGGGATTGATGTGTGGGAGCACG	AGACAGGACGTTATCTTGCTGGGA
Runx2	GCCGTAGAGAGCAGGGAAGAC	CTGGCTTGGATTAGGGAGTCAC
GAPDH	AGAAAAACCTGCCAAATATGATGA	CTGGGTGTCGCTGTTGAAGTC

### Statistical evaluation

All the quantitative data used in this study were analyzed. One-way analysis of variance (one-way ANOVA) was used for multiple group comparisons followed by Tukey’s post hoc test and expressed as the means ± SD with *n* ≥ 3. Significance was measured at the following thresholds: *compared to the Control or Sham group, the difference was statistically significant at *p* < 0.05; ^#^compared to the Selenium or H_2_H_2_ group, the difference was statistically significant at *p* < 0.05; ^&^in comparison with TUDCA or H_2_H_2_ + Se, the difference was statistically significant at *p* < 0.05; ^&^In comparison with H_2_H_2_+ TUDCA, the difference was statistically significant at *p* < 0.05.

## Results

### Se/TUDCA promotes bone repair of distal femur in osteoporotic rats

**Fig. 1:**
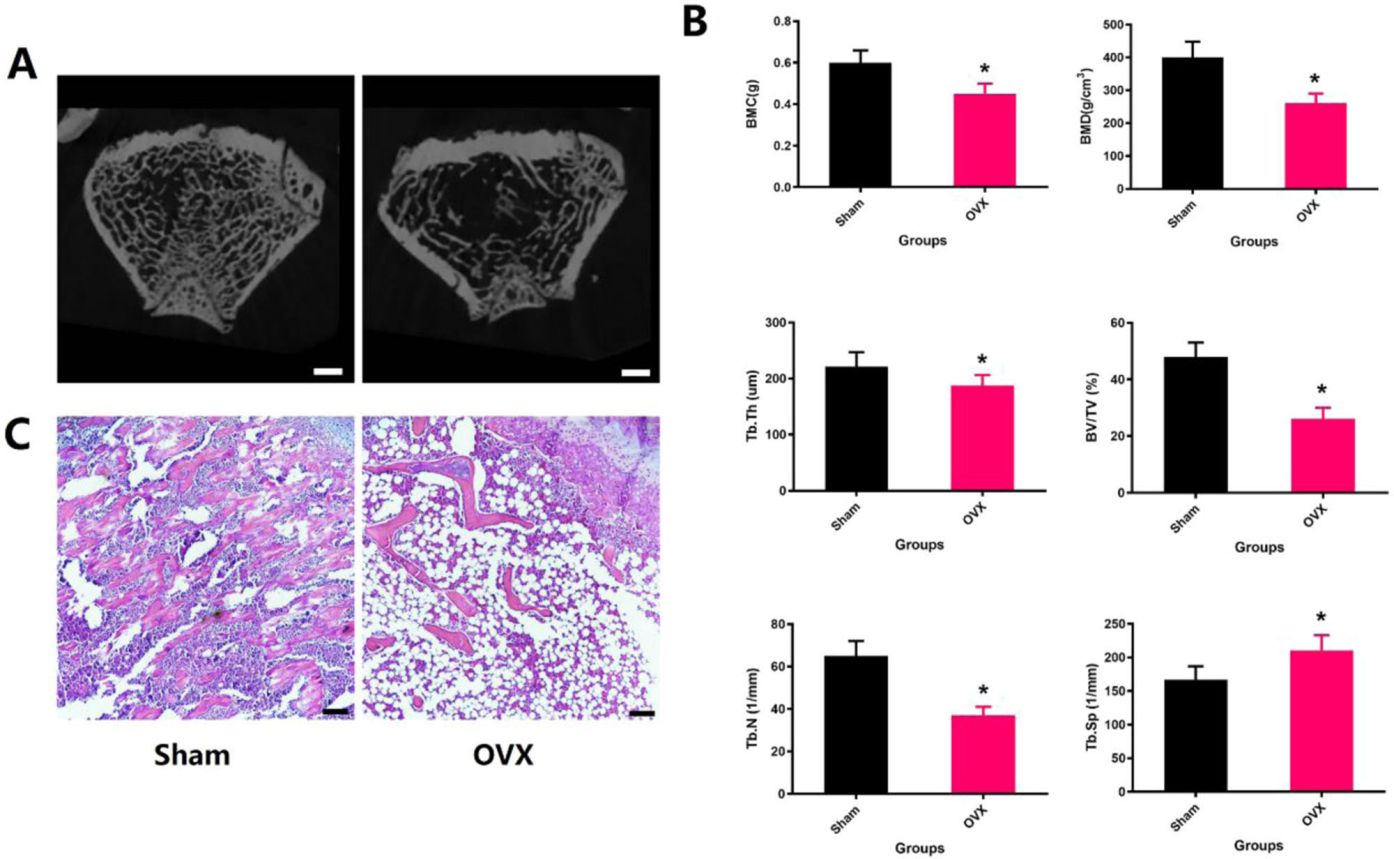
Extensive bone loss and reduced bone mineral density in the model of the ovariectomized rat. **A** Representative images of the bone trabeculae around the epiphyseal line of the distal femur were scanned by Mciro-CT, suggesting that OVX could significantly reduce trabecular loss (scale bar = 1 mm). **B** Bone trabeculae around the epiphyseal line of the distal femur were scanned by Mciro-CT and denoted as BMD, BMC, BV/TV, Tb.N, Tb.Th and Tb.Sp. **C** The distal femur was stained with HE, and a large area of osteoderms around the epiphyseal line was observed bone trabeculae in the OVX group, while the same part of the Sham group was found to be filled with bone trabeculae (magnification of 10)

To verify the effect of Se/TUDCA on bone repair of distal femur in osteoporotic rats, Micro-CT and HE were performed to detect the BMD, BMC and trabecular bone microstructure of bone defect and changes of trabecular bone in distal femur. The BMD and BMC of the distal femur were found to be significantly reduced in the OVX rats compared to the Sham group (*P* < 0.05, Fig. 1). Micro-CT analysis revealed that the trabecular bone microstructure in OVX rats exhibited lower BV/TV, Tb.Th, Tb.N and higher Tb.Sp when compared to the Sham group (*P* < 0.05, Fig. 1). Histopathological examination using HE staining showed notable changes in bone loss within the distal femoral region of each group. Specifically, observations under a microscope revealed empty bone marrow spaces, increased presence of fatty cells, and fractures in trabecular bones within the OVX group (Fig. 1).

**Fig. 2:**
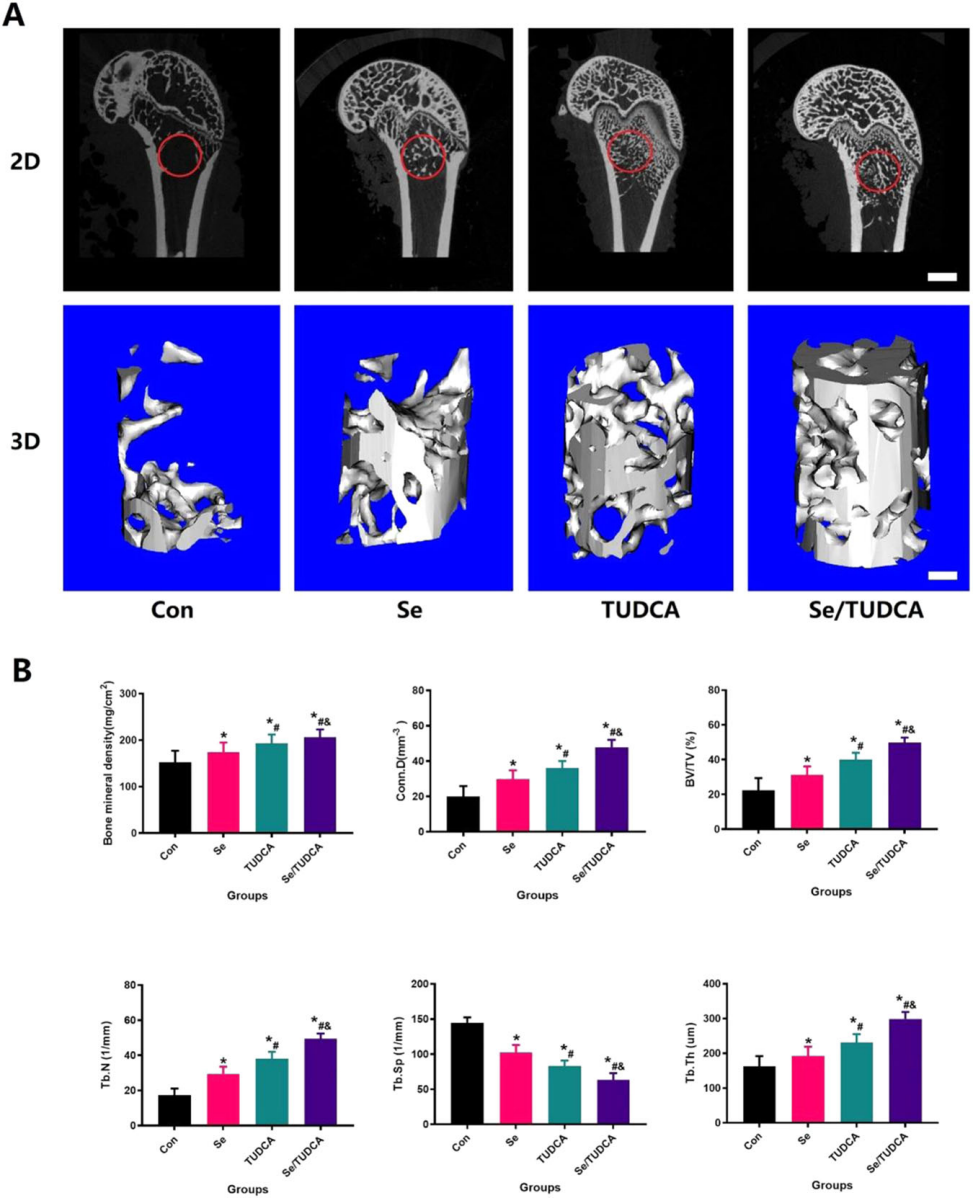
Bilateral ovariectomized rats showed impaired bone repair, and Se/TUDCA were able to significantly improve the bone defect repair potential in OVX rats. **A** Representative images of bone defects in the distal femur were scanned and 3D reconstructed by Mciro-CT, suggesting that Se/TUDCA could significantly improve bone defect repair potential in OVX rats; the red circles represent areas of bone defect (scale bar = 2 mm). The red circles represent the distal bone defects of the femur. **B** New bone tissue surrounding the bone defect in the distal femur was scanned by Micro-CT and denoted as BMD, BV/TV, Tb.N, Conn.D, Tb.Th and Tb.Sp

**Fig. 3:**
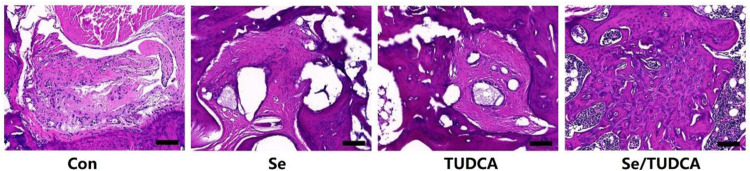
Representative HE images of bone regeneration of defected areas; (magnification, ×10)

The imaging and histology results revealed that the OVX group experienced significant hindrance in bone regeneration, resulting in large areas of remaining bone defects. Conversely, the Se, TUDCA and Se/TUDCA treatment groups exhibited mostly filled new bone tissue within the bone defects (Figs. 2, 3). Notably, when comparing the Se/TUDCA group with TUDCA and Se intervention, superior repair of bone defects was observed (Figs. 2, 3). Micro-CT evaluation demonstrated that rats treated with Se/TUDCA displayed higher BMD, BV/TV, Tb.Th, Tb.N and lower Tb.Sp compared to both the Se and TUDCA groups (*P* < 0.05; Fig. 2).

### Se/TUDCA regulate the activity of osteoblast and osteoclast in osteoporotic rats

**Fig. 4:**
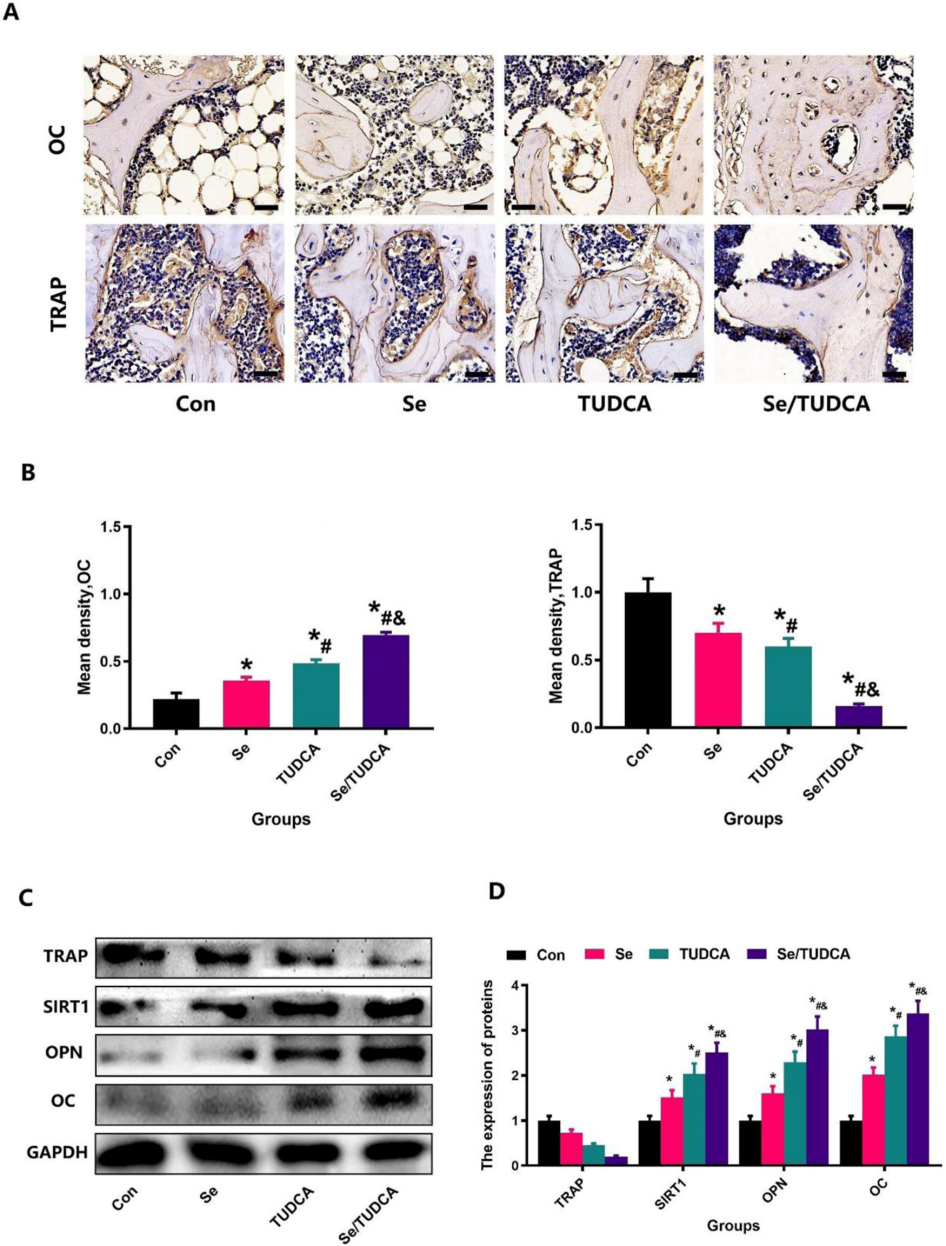
The status of bone reconstruction in the distal femoral defect region of OVX rats was observed by immunohistochemical staining. **A** After Se/TUDCA treatment, the defective area was detected by OC staining (magnification, ×63) and TRAP staining (magnification, ×63) in the four treated-groups. **B** After Se, TUDCA and Se/TUDCA treatment, the density of the OC- and TRAP-stained area was quantified. **C** After treatment, related protein expression in the defective area was detected by WB in the four treated-groups. **D** After Se, TUDCA and Se/TUDCA treatment, the density of the related protein expression was quantified

**Fig. 5:**
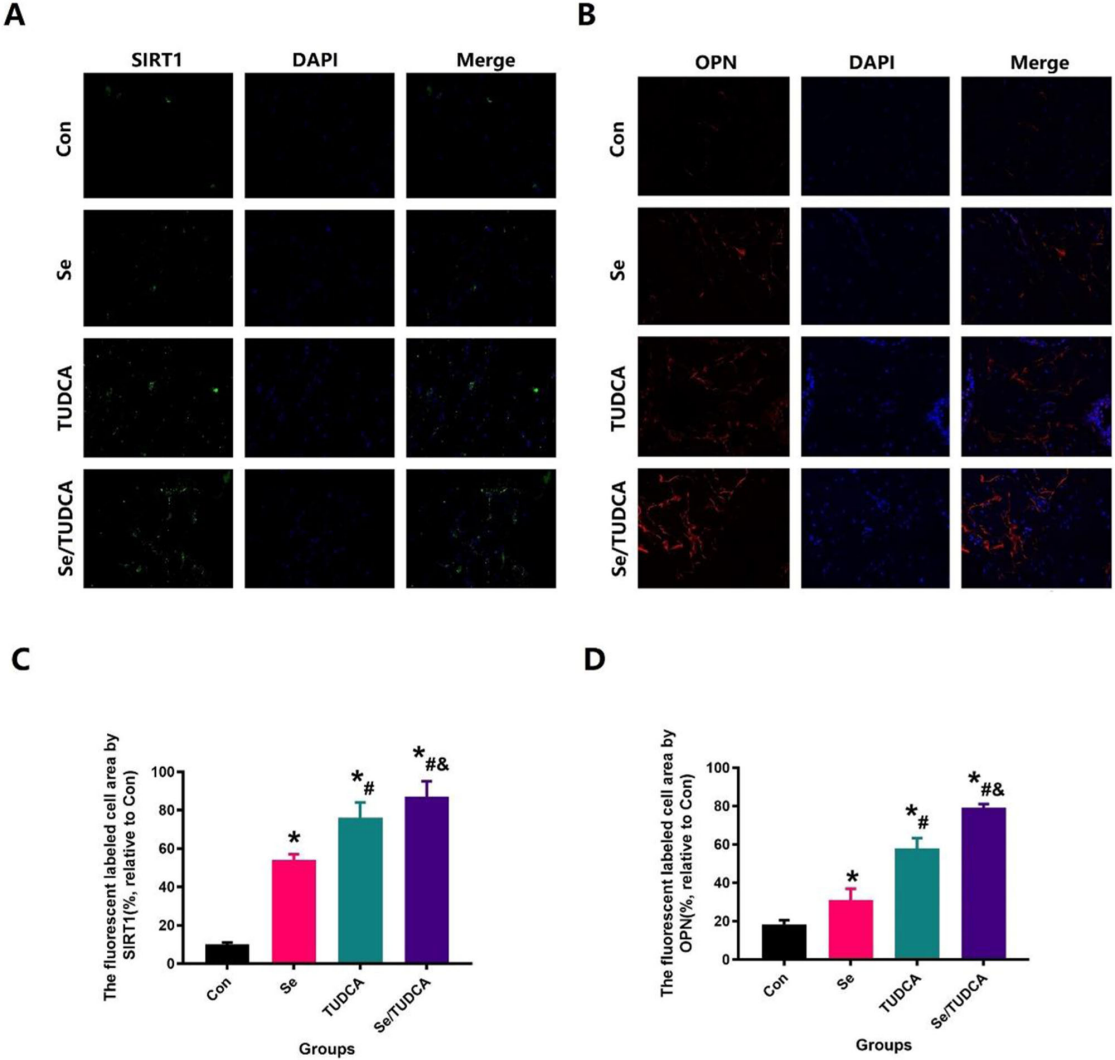
The levels of SIRT1 and OPN in the distal femoral defect region of OVX rats were observed by immunofluorescence staining. After Se, TUDCA and Se/TUDCA treatment, the defective area was detected by SIRT1 staining (magnification, ×63; **A**) and OPN staining (magnification, ×63; **B**) in the three groups. **C**, **D** The density of the SIRT1- and OPN-stained area was quantified

To verify the effect of Se/TUDCA in osteoblasts and osteoclasts among OVX rats, we performed TRAP, OPN and OC immunofluorescence and immunohistochemical staining (Figs. 4A, 5B). Our findings indicate that treatment with Se and TUDCA significantly reduced the expression of TRAP, OPN and OC compared to the control group (*P* < 0.05). Additionally, rats administered with Se/TUDCA displayed notably lower levels of TRAP, OPN and OC expression when compared to both the Se group and TUDCA group (*P* < 0.05, Fig. 4B). The findings from the WB analysis provided additional evidence that Se/TUDCA treatment led to a significant decrease in the levels of TRAP, OPN and OC proteins in bone defect area, as compared to both the Se group and TUDCA group (*P* < 0.05, Figs. 4C, D and 5B, D).

### Se/TUDCA reduces oxidative stress in osteoporotic rats

Previous studies have shown that activation of the SIRT1 signaling pathway can suppress oxidative stress [28, 29, 35]. To verify the role of Se/TUDCA on SIRT1 in OVX rats, SIRT1 staining was performed to detect changes in oxidative stress (Fig. 5A, C). Our results showed that Se, TUDCA and Se/TUDCA treatment significantly increased the fluorescence intensity of SIRT1 in bone tissue, compared with the control group (*P* < 0.05). Besides, Se/TUDCA-treated rats significantly increased the fluorescence intensity of SIRT1 in contrast to the Se and TUDCA groups (*P* < 0.05, Fig. 5C). The findings from the WB analysis provided additional evidence that Se/TUDCA treatment led to a significant increase in the levels of SIRT1 protein in bone defect area, as compared to both the Se group and TUDCA group (*P* < 0.05, Fig. 4C, D)

### Se/TUDCA promotes proliferation and differentiation of osteoblasts

**Fig. 6:**
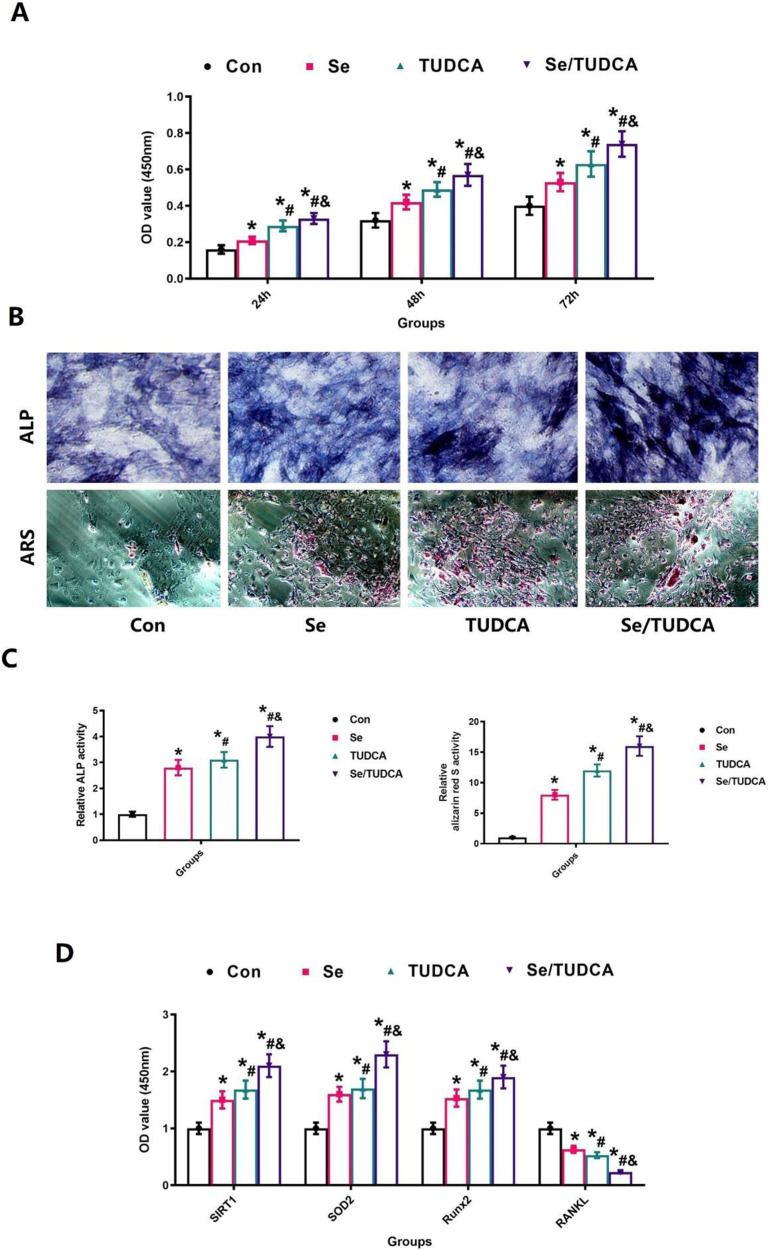
Se, TUDCA and Se/TUDCA treatment can significantly promote osteoblast proliferation and expression of related genes. **A** The results of CCK-8 of MC3T3-E1 under different intervention conditions; **B** Se, TUDCA and Se/TUDCA therapy increased MC3T3-E1 osteogenic potential. **C** The changes in ALP expression and the ability to form calcified nodules in MC3T3-E1 after Se, TUDCA and Se/TUDCA intervention were quantitatively assessed. **D** Quantitative results on the expression of relevant genes were quantitatively assessed after Se, TUDCA and Se/TUDCA intervention

To explore whether Se/TUDCA can promote osteoblast proliferation, differentiation and biological function by inhibiting oxidative stress, we explored different interventions in osteoblast progenitor cells MC3T3-E1 and observed their effects on biological function. The CCK-8 experiment demonstrated that treatment with Se, TUDCA and Se/TUDCA significantly enhanced cellular proliferation. Notably, the combination of Se and TUDCA exhibited the most potent effect (Fig. 6A). To evaluate the visual effects of Se/TUDCA on osteogenesis, we conducted a study to examine their influence on the differentiation process of MC3T3-E1. Our experimental findings indicate that both Se and TUDCA, as well as their combination (Se/TUDCA), significantly promote the differentiation of MC3T3-E1 cells into bone-forming cells. This is supported by a noticeable increase in ALP expression and improved ability to form calcified nodules (*P* < 0.05, Fig. 6B, C). Importantly, the group treated with Se/TUDCA demonstrated superior performance (*P* < 0.05, Fig. 6B, C). We also observed the expression of relevant genes, namely SIRT1, RANKL, SOD2 and Runx2, which exhibited consistency with ALP activity and the formation of calcified nodules. The administration of Se, TUDCA and Se/TUDCA all resulted in a significant decrease in RANKL expression while upregulating the expression of SIRT1, SOD2 and Runx2. Notably, the most pronounced changes in gene expression were observed following intervention with Se/TUDCA (*P* < 0.05, Fig. 6D).

### Se/TUDCA can resist oxidative stress and promote osteogenesis by activating SIRT1 signaling

**Fig. 7:**
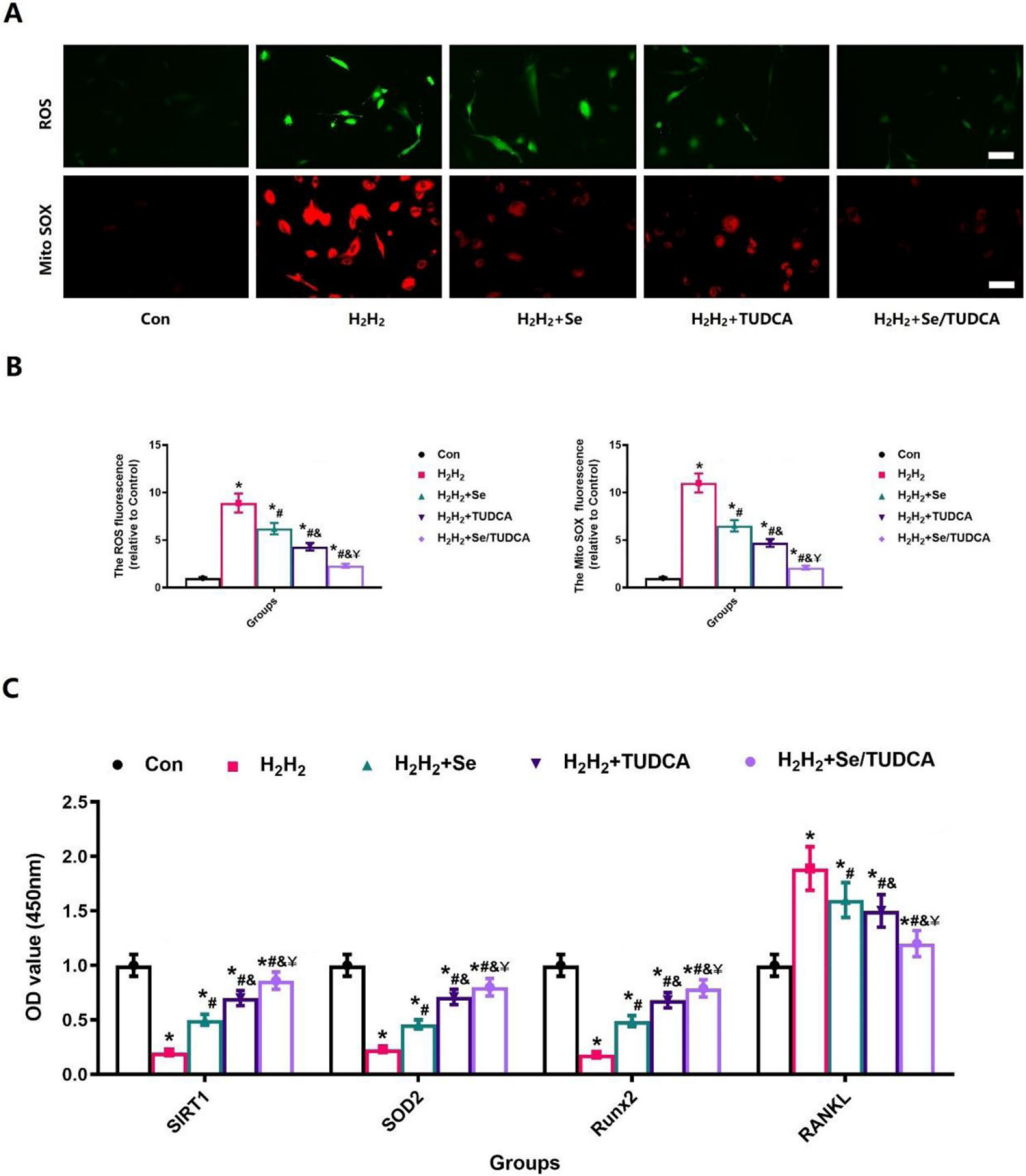
Se, TUDCA and Se/TUDCA therapy increased the MC3T3-E1 anti-oxidative stress potential in H_2_H_2_ treated Conditions. **A** Se, TUDCA and Se/TUDCA therapy reduces the intracellular oxidative stress that causes damage in MC3T3-E1 due to high oxidative stress environment as assessed by ROS and Mito SOX in H_2_H_2_ treated conditions (scale bar = 25 µm); **B** ROS and Mito SOX fluorescence staining intensity of MC3T3-E1 cells in each group; **C** Quantitative results on the expression of relevant genes were quantitatively assessed after Se, TUDCA and Se/TUDCA intervention in H_2_H_2_ treated conditions

The correlation between osteoporosis and increased levels of oxidative stress has motivated our exploration into the potential benefits of Se/TUDCA in enhancing osteogenic differentiation and reducing oxidative stress, owing to their remarkable abilities in combating oxidative stress. The induction of high levels of oxidative stress using H_2_H_2_ revealed that Se, TUDCA and Se/TUDCA all exhibited significant reductions in reactive oxygen species (ROS) and Mito SOX levels through the detection of cellular ROS and mitochondrial ROS (Mito SOX) (*P* < 0.05, Fig. 7A, B). Notably, Se/TUDCA demonstrated the most pronounced effect in reducing both ROS and Mito SOX levels (*P* < 0.05, Fig. 7A, B). The administration of Se, TUDCA and Se/TUDCA resulted in a significant decrease in RANKL expression while simultaneously increasing the expression of SIRT1, SOD2 and Runx2 under conditions characterized by high levels of oxidative stress. Notably, the most prominent alterations in gene expression were observed when using Se/TUDCA intervention during periods of elevated oxidative stress (*P* < 0.05, Fig. 7C).

**Fig. 8:**
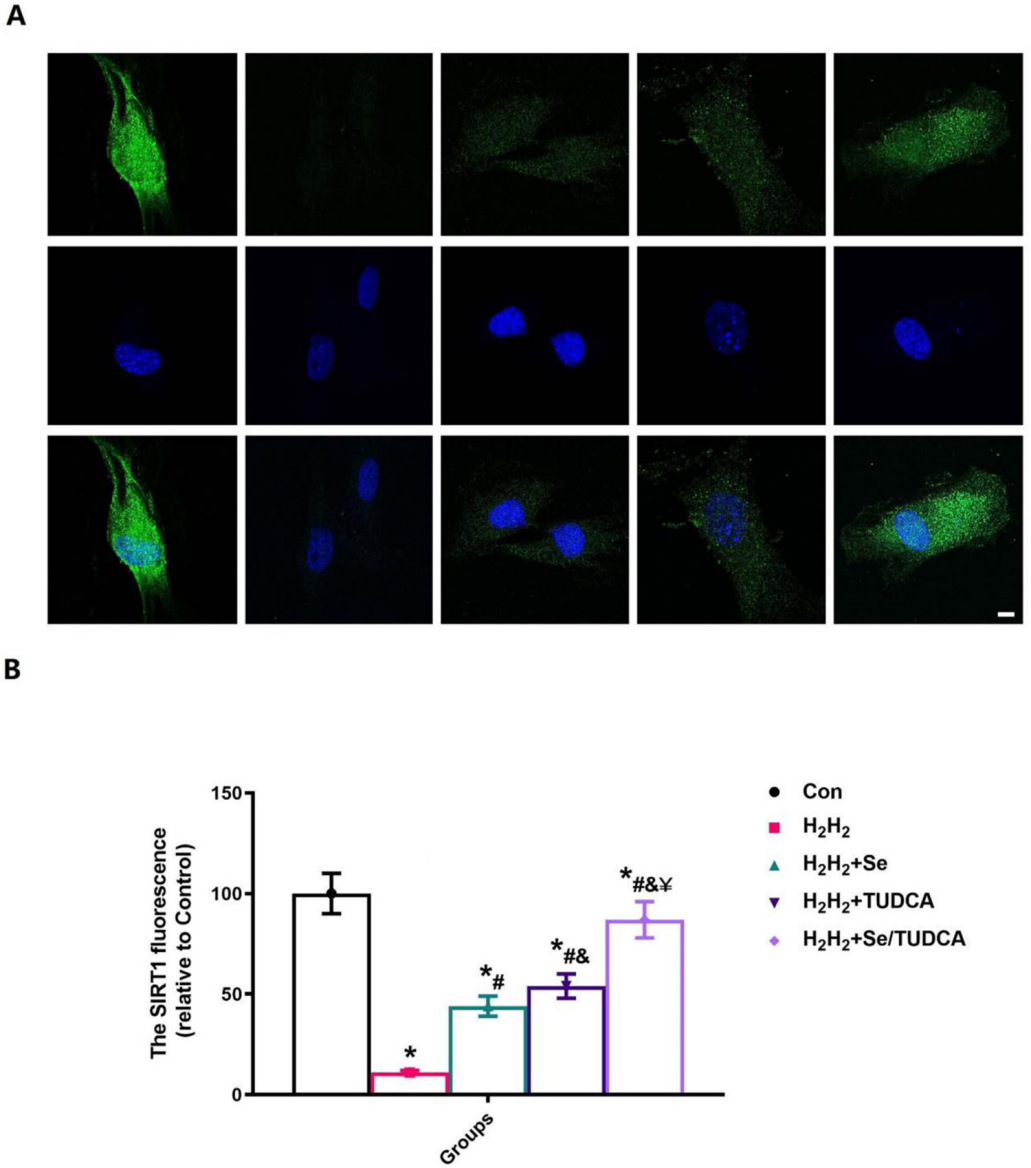
Se, TUDCA and Se/TUDCA therapy increased the expression of SIRT1 of the MC3T3-E1 in H_2_H_2_ treated conditions. **A** SIRT1 was observed at high magnification to assess the ability of Se, TUDCA and Se/TUDCA to significantly improve the resist oxidative stress in H_2_H_2_ treated-MC3T3-E1 (scale bar = 25 µm); **B** Quantitative analysis of changes in SIRT1 expression in H_2_H_2_ treated-MC3T3-E1 after Se, TUDCA and Se/TUDCA interventions

The treated cells were further subjected to SIRT1 immunofluorescence staining, as SIRT1 plays a crucial role in the regulation of osteoblastic activity and resistance to oxidative stress [28, 29, 35]. The increase of SIRT1 fluorescence intensity visually showed that Se, TUDCA, and Se/TUDCA treatment could significantly increase the cellular antioxidant stress ability (*P* < 0.05, Fig. 8). Although Se and TUDCA also inhibit oxidative stress in cells and significantly increase the biological function of osteoblasts, the effect of inhibiting oxidative stress is less pronounced than that of Se/TUDCA (*P* < 0.05, Fig. 8). The findings suggest that Se/TUDCA enhances osteoblast proliferation, differentiation and function by activating the SIRT1 signaling pathway.

## Discussion

Non-union or delayed healing of osteoporosis fractures and defects poses great challenges for orthopedic surgeons and patients. Bone regeneration is a complex process involving the activation of osteoblasts, the formation of new bone matrix and the remodeling of existing bone tissue. Studies have shown that TUDCA can promote osteoblast differentiation and mineralization, both of which are essential processes in bone formation [23]. By enhancing the activity of osteoblasts, the cells responsible for building new bone, TUDCA has the potential to accelerate bone regeneration and healing [23]. Se, in its various forms, interacts with various bone-related proteins and enzymes, enhancing their functionality and promoting bone formation [20]. For instance, Se-containing proteins, such as selenoprotein P, have been shown to stimulate osteoblast proliferation and differentiation, thereby promoting bone growth and repair [21]. In light of the potential of TUDCA and Se in promoting bone regeneration for osteoporosis, our objective was to investigate the synergistic effect of combining TUDCA and Se. We have conducted preliminary animal and cell experiments, which suggest that the combination of TUDCA and Se may have the potential to enhance bone regeneration in osteoporosis.

In this study, we validated the effect of TUDCA, Se and Se/TUDCA on promoting bone repair by comparing bone formation at the defect site after implantation in OVX rats. Histological and Micro-CT analysis showed that significant new bone tissue formation was observed in the bone defect sites in treatment groups at 12 weeks post-implantation. Treatment with Se/TUDCA resulted in higher bone formation compared to treatment with TUDCA or Se, suggesting that Se/TUDCA is a more effective bone formation stimulator than TUDCA or Se in OVX rat regeneration. To further observe the metabolic status of bone tissue in the defect region, immunohistochemistry experiments were performed to detect OC, OPN and TRAP expression in bone tissues from defected area specimens [36]. In the control group, the fluorescence intensity of OC, OPN and TRAP was upregulated in the bone tissue of the defect region, indicating the cause of the blocked bone repair in osteoporosis. In the Se, Se/TUDCA and TUDCA treatment groups, expression of OC, OPN and TRAP was suppressed at new bone masses, whereas Se/TUDCA treatment induced more bone tissue formation and significantly decreased high bone turnover. Therefore, it was not surprising that Se, Se/TUDCA and TUDCA therapy showed favorable results in promoting bone formation.

Lastly, oxidative stress, a separate risk factor for osteoporosis, is increasingly being considered in osteoporosis [37–39]. Moreover, the imbalance in oxidants and antioxidants contributes to osteoclasts inducing bone resorption and osteoblasts mediating bone formation [37–39]. Recent studies have suggested that TUDCA can be used to maintain homeostatic levels of ER stress in cells. TUDCA and Se all could resist imperfect differentiation and inflammation of stem cells due to extracellular changes [40, 41]. Since postmenopausal osteoporosis may be closely related to elevated levels of oxidative stress, previous studies have confirmed that Se and TUDCA have protective effects on bone impairment due to oxidative stress [40, 41]. We, therefore, used immunofluorescence staining to observe the activity of SIRT1 [35, 42], a specific marker of oxidative stress in bone tissue. In the control group, the fluorescence expression intensity of SIRT1 was suppressed in the bone tissue of the defect region, indicating the anti-oxidative stress ability of bone tissue decreased in osteoporosis. In the Se, Se/TUDCA and TUDCA treatment groups, the expression of SIRT1 was upregulated in the original bone formation at the insertion site, while the Se/TUDCA treatment accelerated higher new bone regeneration against oxidative stress.

During bone remodeling, the differentiation and function of osteoblasts play a crucial role in bone formation and maintain bone metabolic homeostasis [43]. Therefore, we performed in vitro studies to observe the effects of Se, Se/TUDCA and TUDCA on osteoblasts, and the results showed stronger effects on osteoblastic proliferation and differentiation. In vitro experiment shows that Se, Se/TUDCA and TUDCA treatment increased ALP expression and calcified nodule formation, representing osteanagenesis activity, and Se/TUDCA treatment obtain the better effect. As shown in Fig. 7, TUDCA and Se treatment reduces oxidative stress in *MC3T3-E1*, representing a reduction in both Mito SOX and ROS, while Se/TUDCA shows a better therapeutic effect on oxidative stress. In addition, SIRT1 activity increased after administration with Se, Se/TUDCA and TUDCA. Based on the obtained results, it is reasonable to conclude that the good effect of Se/TUDCA in osteoporotic rats with distal femoral defects may be directly related to its strong anti-oxidative stress effect, powerful upregulation of osteoblast function and promotion of osteogenic differentiation [44].

## Conclusion

Given the higher incidence of bone non-union in osteoporotic conditions, we attempted to explore the feasibility of Se/TUDCA as a potent inducer in osteoporotic bone defect models. Comparative studies with Se and TUDCA demonstrated a strong positive effect of Se/TUDCA on osteoporotic bone defect regeneration in vivo and in vitro. At the same time, preliminary observations of Se/TUDCA in the field of anti-oxidative stress and its role in promoting bone regeneration may be relevant.
